# Honey Bees Avoid Nectar Colonized by Three Bacterial Species, But Not by a Yeast Species, Isolated from the Bee Gut

**DOI:** 10.1371/journal.pone.0086494

**Published:** 2014-01-22

**Authors:** Ashley P. Good, Marie-Pierre L. Gauthier, Rachel L. Vannette, Tadashi Fukami

**Affiliations:** Department of Biology, Stanford University, Stanford, California, United States of America; Royal Holloway University of London, United Kingdom

## Abstract

The gut microflora of the honey bee, *Apis mellifera*, is receiving increasing attention as a potential determinant of the bees’ health and their efficacy as pollinators. Studies have focused primarily on the microbial taxa that appear numerically dominant in the bee gut, with the assumption that the dominant status suggests their potential importance to the bees’ health. However, numerically minor taxa might also influence the bees’ efficacy as pollinators, particularly if they are not only present in the gut, but also capable of growing in floral nectar and altering its chemical properties. Nonetheless, it is not well understood whether honey bees have any feeding preference for or against nectar colonized by specific microbial species. To test whether bees exhibit a preference, we conducted a series of field experiments at an apiary using synthetic nectar inoculated with specific species of bacteria or yeast that had been isolated from the bee gut, but are considered minor components of the gut microflora. These species had also been found in floral nectar. Our results indicated that honey bees avoided nectar colonized by the bacteria *Asaia astilbes*, *Erwinia tasmaniensis*, and *Lactobacillus kunkeei*, whereas the yeast *Metschnikowia reukaufii* did not affect the feeding preference of the insects. Our results also indicated that avoidance of bacteria-colonized nectar was caused not by the presence of the bacteria *per se*, but by the chemical changes to nectar made by the bacteria. These findings suggest that gut microbes may not only affect the bees’ health as symbionts, but that some of the microbes may possibly affect the efficacy of *A. mellifera* as pollinators by altering nectar chemistry and influencing their foraging behavior.

## Introduction

Factors affecting the health and efficacy of the honey bee *Apis mellifera* as pollinators are of considerable interest because of their agricultural importance [1]. One potential factor that is receiving increasing attention is bee gut microflora [2]. Described as one of the greatest unexplored reservoirs of microbial diversity [3], the insect gut carries a diverse assemblage of symbiotic bacteria [4]–[6]. For example, species belonging to genera of lactic acid bacteria, such as *Lactobacillus* and *Bifidobacterium*, are frequently found in the honey bee gut and may defend the host against pathogens [7]. Similarly, acetic acid bacteria such as those from the genus *Asaia* and *Gluconobacter* have been indicated as facultative symbionts of honey bees and other sugar-feeding insects [8]–[11], and might also be beneficial to the host through suppression of pathogenic bacteria.

Studies on the bee gut microflora have primarily focused on the taxa that appear numerically dominant in the gut, with the assumption that the dominant status as symbionts suggests that they are particularly important to the bees’ health. However, numerically minor taxa in the gut, including both bacterial and yeast species, might also influence the bees’ efficacy as pollinators. This possibility may be especially likely if the microbes are not only present in the gut, but also capable of growing in bee food resources, including floral nectar, and altering its chemical properties. For example, aerobic species of bacteria and yeast may be found only as minor members of the microflora in the bee gut [12], which can be low in oxygen availability [12], [13], but may attain high abundance in floral nectar. These microbes may affect the chemical properties of nectar and, consequently, its attractiveness to insect pollinators [14]–[17].

Recent work has suggested that the effects of microbial growth in nectar on pollinator preference can differ among microbial species, at least when bumblebees or hummingbirds are the pollinators [15]–[17]. However, it is poorly known whether honey bees have feeding preferences for specific microbes in nectar [18]. As a first step toward answering this question, we conducted a series of field experiments at a small apiary in California. Our aim was to test the hypothesis that microbial growth in nectar affects nectar preference of honey bees, depending on the species identity of the bacteria and yeast. In order to test this hypothesis, we first isolated and identified potential bee-associated microbes that were able to grow in nectar and used some of these isolates in the field experiments.

## Materials and Methods

### Study Site

**Figure 1:**
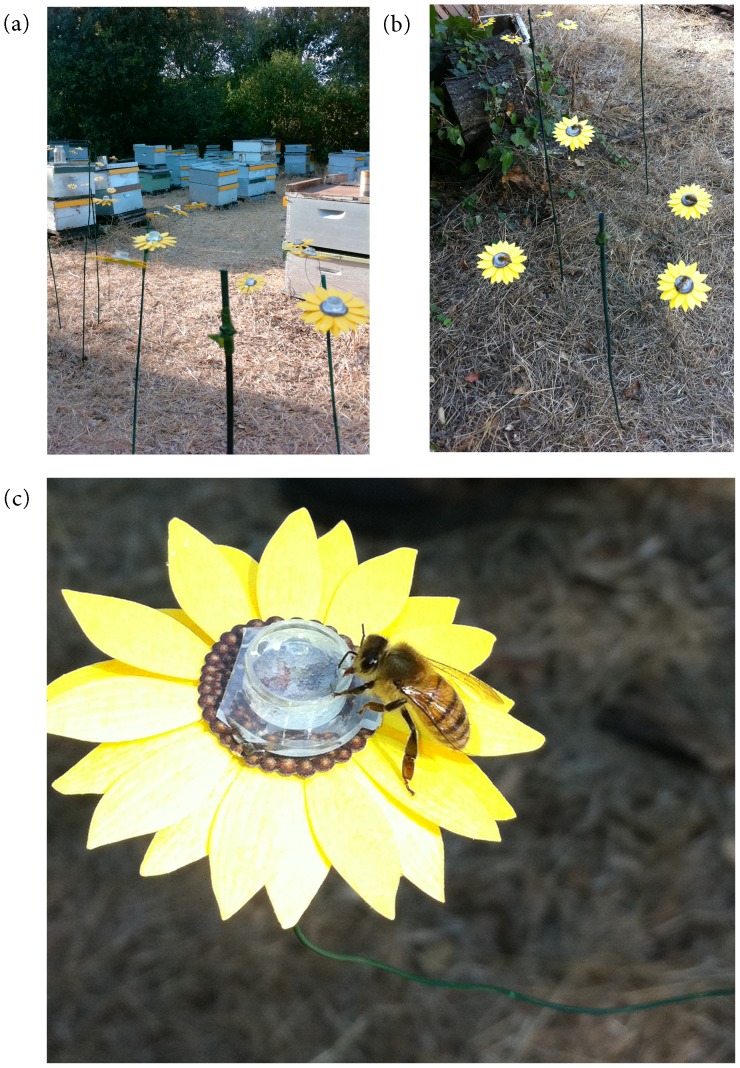
Experimental apiary and artificial flower arrays. (**a**) Apiary with an artificial flower array arranged in the shade, 1–2 meters from hives. (**b**) Experimental flower stand on which honey bees can be seen feeding from flowers containing synthetic nectar inoculated with microbes. Each treatment was represented once on each stand. (**c**) Experimental flower showing a honey bee feeding on synthetic nectar.

The study was conducted at the plant growth facility on the Stanford University campus, located in the San Francisco peninsula of California. This site had a small apiary consisting of approximately 160 honey bee hives (Figure 1a).

### Microbial Sampling from the Bee Gut

To obtain some of the microbial strains from the bee gut, a total of 150 honey bees were captured at the apiary using yellow containers filled with soapy water to trap live bees. For the first field experiment (see *Experimental design* below), we placed 10 containers around the hives at 10∶00 AM and retrieved 55 live bees trapped in the containers at 3∶30 PM on February 29, 2012. We used 50 of the 55 bees for microbial sampling. For the second and third field experiments (see *Experimental design* below), we placed 20 containers at 8∶00 AM and retrieved 80 live bees at 1∶00 PM on June 29, 2012. In addition, we placed 8 containers at 10∶30 AM and retrieved 60 live bees at 1∶00 PM on June 30, 2012. Of the approximately 140 bees trapped on these two days, we used 100 for microbial sampling.

Immediately after removal from the containers, the live bees were placed in a 4°C refrigerator for 3 minutes to slow movement. The bees were then dissected transversely to detach the sting, open the posterior segment of the abdomen, and remove the fully intact intestine containing the gut. Within the gut we sampled the crop, a central organ in the honeybee’s food production, located between the oesophagus and ventriculus and used for collection and transport of nectar to the hive. The contents of each crop were then placed on three replicates of two types of plates (six plates per gut sample): (1) yeast–malt agar (YMA; Difco, Sparks, MD, USA) supplemented with 100 mg/l ampicillin to prevent bacterial growth, but allow yeast growth, and (2) YMA supplemented with 100 mg/l cycloheximide to prevent yeast growth, but allow bacterial growth. Previous studies used similar media to isolate microbes from bee gut [19], [20].

The plates were incubated at 25°C for 3 to 5 days in aerobic conditions. We note that bacteria and yeast found in the honey bee gut that require anaerobic conditions will not have survived under these conditions. The plates were not all incubated for the same length of time, because the length of time required for colony growth seemed variable among species and we were interested in isolating multiple species. For each gut sample, at most three replicates from morphologically distinct colonies were sub-streaked on the plates. Samples from all bees were then pooled in order to identify common colony morphotypes. Up to three replicates of each distinct morphotype were chosen for DNA extraction and amplification with the Sigma RED Extract-N-Amp tissue PCR kit (Sigma-Aldrich, Inc., Saint Louis, MO, USA), which was used according to the manufacturer’s specifications. A portion of the 16S rRNA gene for bacteria and the 18S gene for yeasts were amplified using bacterial primers, U519F and U1099R [21], and fungal primers, NL1 and NL4 [22]. Amplicons were then sequenced by the Stanford University Protein and Nucleic Acid Facility, using an ABI-3130×l Genetic Analyzer (Life Technologies, Carlsbad, CA).

The consensus sequences were grouped into operational taxonomic units (OTU) based on 98% similarity, using Geneious Pro (Biomatters Ltd., Auckland, New Zealand). Consensus sequences of each OTU were identified using Basic Local Alignment Search Tool (BLAST) searches against the National Center for Biotechnology Information’s GenBank. A total of 16 species (14 bacterial and 2 fungal) from the 150 gut dissections were retrieved (Table 1, Figure S1). Of these, strains of commonly found species were kept on YMA and freshly streaked 2–4 days prior to each of the field experiments described below.

**Table 1:** Taxonomic assignments of microorganisms isolated from *A. mellifera* gut specimens in this study.

Taxonomic group	Species	% max identity	BLAST corresponding accession number	GeneBank accession number
Fungi	*Metschnikowia reukaufii*	99	DQ437075.1	KC677750
	*Aureobasidium pullulans*	99	JX303663.1	KC677741
Acetic acid bacteria	*Asaia astilbes*	100	AB485744.1	KC677740
	*Gluconobacter* sp.	99	AB511061.1	KC677748
Lactic acid bacteria	*Fructobacillus fructosus*	99	AB680098.1	KC677747
	*Lactobacillus kunkeei*	97	JQ009353.1	KC677749
Other bacteria	*Acinetobacter boissieri*	99	JQ771141.1	KC677738
	*Acinetobacter nectaris*	99	JQ771134.1	KC677739
	*Brenneria quercina*	100	NR041975.1	KC677742
	*Chryseobacterium* sp.	99	JX437140.1	KC677743
	*Chryseobacterium ureilyticum*	99	JX100826.1	KC677744
	*Enterobacter cloacae*	99	HQ888762.1	KC677745
	*Erwinia amylovora*	99	DQ059817.1	KC677746
	*Erwinia tasmaniensis*	99	NR074869.1	KC677753
	*Micrococcus* sp.	100	JX437142.1	KC677751
	*Rhodococcus kroppenstedtii*	100	EU977670.1	KC677752

### Experimental Flowers

For experiments at the apiary, 40 artificial flowers were handcrafted. The flowers were designed to encourage honey bee visits [23] and consisted of yellow sunflower-shaped paper with a 1.5 ml centrifuge tube cap attached to the center (Figure 1b, c). We attached four of the flowers to each of 10 green-painted bamboo sticks using florist’s wire and green florist’s tape. The sticks were approximately 1 m tall. At the apiary, the 10 stands had 4 flowers with each containing one of the four treatments detailed below. The stands were placed approximately 1–2 m away from the hives (Figure 1a).

### Experimental Design

Using the artificial flowers, three experiments were conducted. In the first two experiments, each vial was filled with 200 µl of synthetic nectar that had been inoculated with: (1) no bacteria or yeast (a control nectar), (2) *Asaia astilbes* (Gram negative bacterium), (3) either *Erwinia tasmaniensis* (Gram negative bacterium) or *Lactobaccillus kunkeei* (Gram positive bacterium), or (4) *Metschnikowia reukaufii* (yeast). When preparing the microbe-inoculated synthetic nectar, we incubated all preparations at 25°C for 4 days prior to each day of each experiment in filter-sterilized 15% w/v sucrose solution supplemented with 0.32 mM amino acids from digested casein. The sucrose and amino acid concentrations were selected so as to mimic typical floral nectar [24]–[26]. Individual colonies of the appropriate species were then diluted to 200 cells per µl each experimental day. Approximately 1.5 ml of this diluted suspension was added to 8 ml of the sucrose solution immediately before the start of the field experiment each day. Therefore, a fresh supply of approximately 32 cells per µl nectar solution was presented to the bees each day of experimentation. Cell densities of 10^4^ yeast cells per µl [27] and 30 bacterial CFU (colony forming units) per µl [17] have been commonly observed in floral nectar in the field. The control nectar was prepared the same way, except that it was not inoculated with microbes. Instead, 1.5 ml of filter-sterilized 15% w/v sucrose solution supplemented with 0.32 mM amino acids from digested casein was added. We added the same amino acids in all treatments including the control because it is well documented that protein type can affect preference in honeybees [28].

In the first experiment, conducted on April 13–15, 2012, *A. astilbes* and *E. tasmaniensis* were used because they appeared to be the most common culturable bacteria in the first set of bee gut specimens. In the second experiment, conducted on September 6–12, 2012, *L. kunkeei* was used instead of *E. tasmaniensis* because *L. kunkeei* was more commonly found in the second set of gut specimens. *M. reukaufii* was used because it is the dominant nectar-inhabiting yeast species in the floral nectar of many plant species [29]–[34] and because we found it in our bee gut specimens as well, even though it is not clear whether or not *M. reukaufii* replicates in the gut.

The third experiment, conducted on September 17–20, 2012, was identical in design to the first two experiments, except that, instead of using *E. tasmaniensis*- or *L. kunkeei*-inoculated nectar as the third treatment group, we used nectar that was inoculated with *A. astilbes* as in the second treatment group, but filter-sterilized (pore size: 0.2 µm) immediately before the experimental use. *A. astilbes* are short rods measuring 0.6 by 1.2–2.0 µm [35]. The filtered nectar treatment was used to determine whether the bees responded to the presence of *A. astilbes* in nectar *per se* or the changes to chemical properties of nectar caused by *A. astilbes*.

During each of the three experiments, new sterile vials containing 200 µl of fresh microbe-inoculated synthetic nectar were used each day. The four flowers on each stand were assigned randomly to one of the four treatment groups each day. Two of the 10 stands were bagged with mesh (mesh size: 1 mm) to deny access by bees in order to account for reduction of nectar weight by evaporation. Approximately two hours after the start of the experiment each day, the remaining nectar from each flower’s vial was capped, brought back to the laboratory, and weighed using a microbalance to estimate changes in volume. Each day, the experiment began at approximately 10∶00 AM. On the hotter days, bees visited the nectar samples more frequently, so we retrieved the samples sooner to have discernible amounts of nectar volume to weigh.

Although we did not directly observe the flowers in the field for the entire experimental period of 2 hours each day, our extensive observations indicated that honey bees were the main, if not the only, animals that visited the flowers (Figure 1). Direct observations of the artificial flowers were conducted during the first 15–30 minutes and the last 10–15 minutes of each of the 2-hour experimental periods. Logistical reasons prevented us from making the direct observations for the entire 2-hour periods. The only other floral visitors that we observed were yellow jackets (*Vespula* spp.), and they were rarely observed (only three times during a week-long experiment) visiting the artificial flowers. Furthermore, even when a yellow jacket landed on an artificial flower, we did not observe any of them consuming the artificial nectar. In contrast, the honey bees were observed frequently visiting and staying on the flowers, and it was possible to see their proboscis in the liquid (Figure 1).

Across the three experiments, ambient temperature at the time of the day the experiment was conducted was approximately 16–25°C, with little to no wind. The area was well shaded for the 1–2 hour experimental window.

### Effect of Microbial Inoculation on Nectar Chemistry

**Figure 2:**
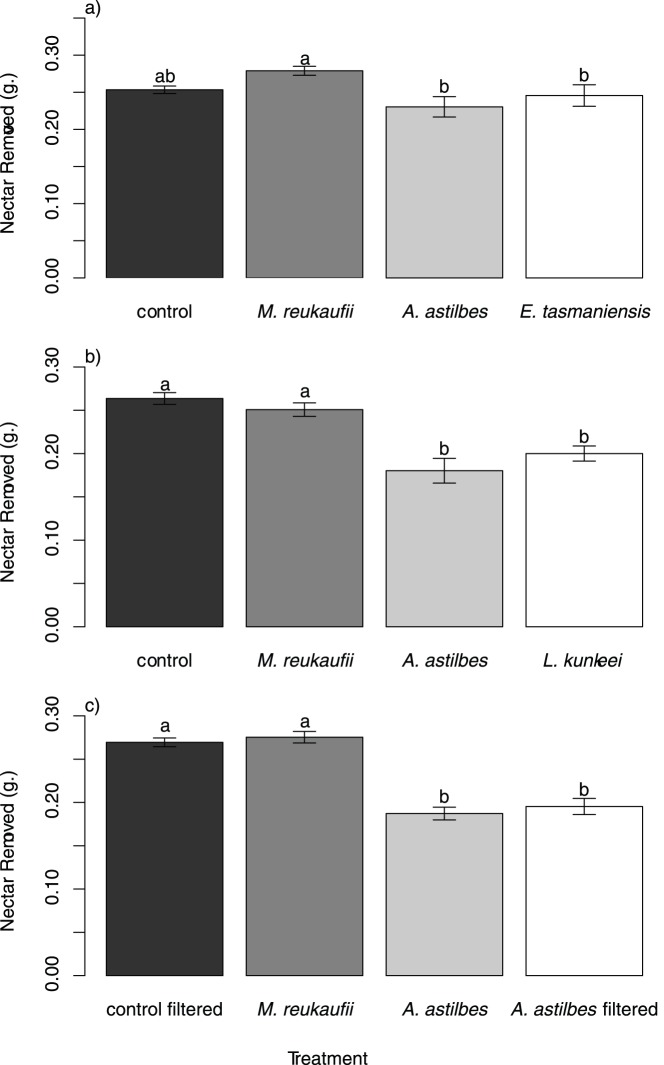
Effects of microbial inoculations on nectar removal by honey bees. (**a**) Results of experiment 1, showing that nectar removal depended on microbial treatment (*Metschnikowia reukaufii*, *Asaia astilbes*, or *Erwinia tasmaniensis*). (**b**) Results of experiment 2, showing that nectar inoculated with *A. astilbes* or *Lactobacillus kunkeei* was removed less than nectar inoculated with yeast (*M. reukaufii*) or no microorganisms. (**c**) Results of experiment 3, showing that nectar inoculated with *A. astilbes* or inoculated with it and then filter-sterilized was removed less than yeast-inoculated and control nectar. Bars indicate means ±1 SE. Letters above bars indicate treatments that differ significantly (Tukey HSD test, α = 0.05).

Because the results of our field experiments indicated that the bees avoided bacteria-colonized nectar not because of the presence of the bacteria *per se*, but owing to the changes in nectar chemistry induced by the bacteria (Figure 2), we conducted an additional experiment in the laboratory to investigate the effect of microbial inoculation on nectar chemistry. To this end, we prepared the microbe-inoculated synthetic nectar exactly as we did for the field experiments, using the same strains of *A. astilbes* and *M. reukaufii* as for the main experiment and a strain of *Erwinia* sp. that we isolated locally from floral nectar of *Mimulus aurantiacus*. In this experiment, we did not use the strain of *L. kunkeei* or *E. tasmaniensis* used in the main experiment, because the stock cultures of these isolates had been lost. After four-day incubation, we measured pH, H_2_O_2_, and sucrose, glucose, and fructose concentrations of the incubated nectar samples, using the methods previously described [17]. We focused on these measurements because previous research indicated that the microbes could induce large changes to these chemical properties and that the changes could have an effect on flower visitors [17], [36]–[38]. A total of 20 experimental units were used, i.e., 4 treatments (control, *A. astilbes*, *M. reukaufii*, and *Erwinia* sp.)×5 replicates.

### Statistical Analysis

The amount of nectar removed by honey bees from each vial was estimated as *a*–*b*, where *a* is the weight (g) of the nectar remaining in the evaporation control vial plus the vial itself after the 2-hour exposure in the field, and *b* is the weight (g) of the nectar remaining in the focal vial plus the vial itself after the 2-hour exposure in the field. We assessed the effects of microbial inoculations on nectar removal using a linear mixed model, with microbial treatment as a fixed effect and experimental day as a random effect, followed by a Tukey HSD test to assess significant differences among treatment levels, using packages nlme [39] and multcomp [40] in R v. 2.15.0 [41]. To examine the variation in treatment effects over time, we also performed one-way analysis of variance (ANOVA) for each day, with the microbial treatments as the predictor variable and the nectar removed as the response variable using R v. 2.15.0 [41]. Within each experiment (experiments 1 to 3), a sequential Bonferroni correction was used to account for multiple tests over several days. ANOVA, followed by a Tukey HSD test, was used to test for the effect of the species inoculated on each chemical measurement.

## Results

In the first and second experiments, 3 to 31% less nectar was removed from experimental flowers when inoculated with *A. astilbes*, *E. tasmaniensis* or *L. kunkeei* than with *M. reukaufii* or with no microorganisms (experiment 1: treatment F_3, 90_ = 6.97, *P*<0.001, Figure 2a; experiment 2 treatment: F_3,214_ = 20.30, *P*<0.0001, Figure 2b). In both experiments, this trend was not significant on the first two days, but subsequently became significant (Figure S2a, b). Similarly, in the third experiment, approximately 32% less nectar was removed when inoculated with *A. astilbes* than with *M. reukaufii* or the control (experiment 3: F_3, 121_ = 43.26, *P*<0.001, Figure 2c). The amount of nectar removed was indistinguishable between the treatment in which *A. astilbes* was inoculated (but not filtered) and the treatment in which *A. astilbes* was inoculated and then filtered (Figure 2c). These trends were consistent throughout the duration of the experiment (Figure S2c).

**Figure 3:**
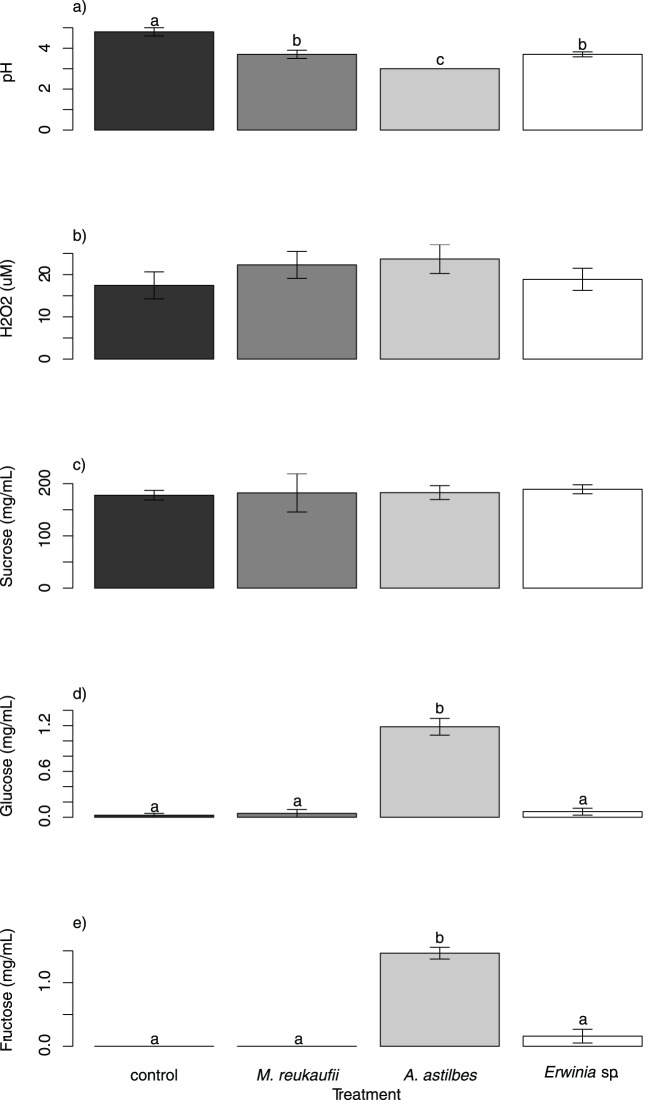
Effects of microbial inoculations on (a) pH, (b) H_2_O_2_, (c) sucrose, (d) glucose, and (e) fructose concentrations in nectar. Bars and letters are as in Figure 2.

In the experiment that investigated the effect of microbial inoculation on nectar chemistry, all three species reduced pH significantly, and *A. astilbes* caused a greater reduction than *M. reukaufii* and *Erwinia* sp. (Figure 3a). No significant difference in H_2_O_2_ or sucrose concentration was detected between any of the treatments (Figure 3b, c). *A. astilbes* increased glucose and fructose concentrations, whereas *M. reukaufii* and *Erwinia* sp. caused no detectable changes (Figure 3d, e).

## Discussion

Taken together, our results provide support for the hypothesis that microbial growth in nectar affects feeding preference of honey bees, and that the effect depends on the identity of microbial species. Specifically, our data indicate that honey bees prefer nectar free of colonies of the three aerobic bacterial species we isolated from the bee gut, whereas the nectar-inhabiting yeast *M. reukaufii* had no effect on bees’ feeding preference. Our data also indicate that avoidance of bacteria-colonized nectar was caused not by the presence of the bacteria *per se*, but by the chemical changes to nectar made by the bacteria. Overall, this study may suggest that gut-inhabiting microbes not only affect the health of *A. mellifera* as symbionts, but potentially also influence their foraging behavior by altering nectar chemistry.

We expected honey bees to prefer nectar inoculated with bacteria because the literature suggested that the bacterial taxa we used could be beneficial as symbionts in the honey bee gut [3], [4], [7]–[9], [42]–[44]. Although evidence for the potential of *E. tasmaniensis* to be an important insect symbiont is not definitive [45], *L. kunkeei* has been indicated to be a mutualistic symbiont of *A. mellifera* [46], and several species of *Asaia* have been indicated as dominant symbionts of some species of insects, e.g., the mosquito *Anopheles stephensi* [8], [9] and the leafhopper *Scaphoideus titanus* [8], [47]. Molecular sequencing indicated that *A. astilbes* and *L. kunkeei* are relatively closely related to one of the clusters of the purported dominant bacteria (the Alpha-2.2 phylotype and the Firm-4 phylotype, respectively) in the honeybee gut [43], but none of our bacterial strains were phylogenetically nested in any of these clusters (Figure S1). Even if our bacterial strains are not numerically dominant in the bee gut, this may not necessarily indicate they are functionally unimportant as symbionts or, as our results now suggest, as modifiers of bee foraging behavior.

The results of the experiment that investigated the effect of microbial inoculation on nectar chemistry suggest that changes in either nectar pH or glucose or fructose concentration could be a reason why bees might avoid bacteria-inoculated nectar. However, although *A. astilbes* and *Erwinia* sp. affected nectar chemistry differently (Figure 3), this difference did not appear to affect nectar removal by bees (Figure 2). Furthermore, it has been indicated that bees prefer sucrose over glucose or fructose when each is offered as a single sugar [48], so increased glucose and fructose might not be a plausible explanation for behavioral choices. In addition, it is intriguing that the measured changes in nectar properties in the *M. reukaufii* and *Erwinia* sp. treatments were similar, yet *M. reukaufii* did not affect foraging whereas *Erwinia* did. One possible explanation for this contrast is that the *Erwinia* sp. used in the experiment to test the effect on nectar properties was functionally different from the *E. tasmaniensis* used in the field experiment on bee preference. Another possibility is that *M. reukaufii* and *Erwinia* spp. had different effects on some important aspects of nectar chemistry that we did not measure. For example, the species may have differed in their ability to produce ethanol. Microbially produced ethanol in nectar has been suggested to alter nectar foraging by insects, e.g., wasps consuming orchid nectar [14]. It is also possible that other volatile organic compounds might play a role [48], but testing these possibilities would require additional experiments.

The capacity of *A. astilbes* to increase glucose and fructose levels without reducing sucrose may seem puzzling (Figure 3). It is likely, however, that the reduction in sucrose concentration that led to the increased glucose and fructose concentration was too small to be detected against the initial variation that existed among replicates within treatments. In contrast, in glucose and fructose, an increase of a similar magnitude could be detected with a higher statistical power because the initial variation in glucose and fructose was essentially non-existent; there was initially no glucose or fructose in our artificial nectar.

Gut microbiota are diverse, containing many more species than the four we focused on in this study [43]. Our purpose was not to characterize the gut microbial community; instead we focused on selected bacterial and yeast strains found in both the bee gut and floral nectar. Our results suggest that it may be worthwhile to investigate the effects of a greater variety of species to evaluate the generality of our findings. Many, though probably not all, of other gut symbiotic species may be capable of growing in nectar. It would be interesting to use different culture conditions, including those that involve lowered O_2_ levels [49], [50], to isolate different strains, including those that belong to the dominant groups of the gut microflora as identified by recent studies [43], [51]–[53], and repeat the bee foraging preference experiment. It is worth noting, however, that more oxygen may normally be available in nectar than in the gut. It is therefore possible that only a subset, if any, of the bacteria that require low O_2_ levels for growth can reproduce in nectar to a sufficient degree to have a large effect on bees. In addition to conducting behavioral tests using single-species inoculations from additional microbial species, setting up multi-species inoculations would be interesting as floral nectar is often likely to contain more than one species. Such work will further advance our understanding of the effect of gut microbes in nectar on bee foraging choices.

If nectar-colonizing bacteria influence flower visits by honey bees, their altered foraging behavior may have consequences for pollination. Although we used a realistic mixture of sugars and amino acids in the synthetic nectar, future studies could use real flowers to confirm the relevance of our findings to pollination by bees. In this context, the contrast in behavioral response we found between the negative effect of bacterial nectar colonization and the neutral effect of yeast nectar colonization is especially intriguing. *M. reukaufii*, which is the most dominant yeast species in nectar in our study region [31] and many other places around the world [29], [30], [32]–[34], has been shown to grow rapidly and reach high density in nectar, subsequently changing the chemical properties of nectar considerably [17], [54], [55]. Even so, this species did not seem to affect bee foraging, whereas the bacteria that we studied did. This finding is consistent with our recent work on nectar consumption by hummingbirds, in which we found that nectar foraging by the birds was reduced as a result of nectar colonization by a bacterium (*Gluconobacter* sp.), but not by *M. reukaufii* [17]. Taken together, our results highlight the importance of studying species-specific effects of microbial colonization in order to understand their potential effects on bee foraging and pollination.

## Supporting Information

Figure S1 **Phylogenetic relationships, based on bacterial 16S rRNA sequences, of the bacterial species used in our experiment and those included in Figures S3B, E, and F of Martinson et al. (2011).**
(DOCX)

Figure S2 **Changes over experimental days in the amount of nectar removed.**
(DOCX)
