# Supplementary material for: Honey Bees Avoid Nectar Colonized by Three Bacterial Species, But Not by a Yeast Species, Isolated from the Bee Gut
Source: PLoS One. 2014 Jan 22;9(1):e86494. doi: 10.1371/journal.pone.0086494 (PMC3899272; doi:10.1371/journal.pone.0086494)
Supplement: Figure S1 — Phylogenetic relationships, based on bacterial 16S rRNA sequences, of the bacterial species used in our experiment and those included in Figures S3B, E, and F of Martinson et al. (2011). (DOCX) [file pone.0086494.s001.docx]

**Figure S1.** Phylogenetic relationships, based on bacterial 16S rRNA sequences, of the bacterial species used in our experiment and those included in FiguresS3B, E, and F of Martinson et al. (2011). All sequences from Martinson et al. (2011) were aligned with recovered sequences from this study using Geneious alignment. Sequences of our species, **(a)** *A. astilbes*, **(b)** *L. kunkeei*, and **(c)** *E. tasmaniensis*, were aligned with all sequences included in Figures S3B, S3E, and S3F of Martinson et al. (2011), respectively. Phylogenetic trees were computed using PHYML using a GTR model and 100 bootstrap replicates (Guindon and Gascuel 2003 *Syst. Biol.* 52:696-704). Numbers on branches represent bootstrap support.

a)

b)

c)
