# Supplementary material for: Honey Bees Avoid Nectar Colonized by Three Bacterial Species, But Not by a Yeast Species, Isolated from the Bee Gut
Source: PLoS One. 2014 Jan 22;9(1):e86494. doi: 10.1371/journal.pone.0086494 (PMC3899272; doi:10.1371/journal.pone.0086494)
Supplement: Figure S2 — Changes over experimental days in the amount of nectar removed. (DOCX) [file pone.0086494.s002.docx]

**Figure S2.** Changes over experimental days in the amount of nectar removed. Asterisks indicate days showing significant preference. **(a)** Results of experiment 1, showing that nectar removal depended on microbial treatment on day 3. **(b)** Results of experiment 2, showing that nectar inoculated with *A. astilbes* or *L. kunkeei* was removed significantly less than nectar inoculated with yeast or no microorganisms, starting day 4. **(c)** Results of experiment 3, showing that both *A. astilbes*-inoculated and *A. astilbes*-filtered nectar were removed less than yeast-inoculated and control nectar, starting day 1. Experiment 3 was started 3 days after experiment 2 ended. Experiment 2 began approximately 5 months after experiment 1 (see the main text for exact dates).
